# Surgical Challenge of Sublobar Resection for Early-stage Non-small Cell Lung Cancer: Current Status and Future Perspectives

**DOI:** 10.14789/ejmj.JMJ25-0062-P

**Published:** 2026-02-10

**Authors:** ARITOSHI HATTORI, TAKESHI MATSUNAGA, MARIKO FUKUI, KAZUYA TAKAMOCHI, KENJI SUZUKI

**Affiliations:** 1 Department of General Thoracic Surgery, Juntendo University School of Medicine, Tokyo, Japan; 1 Department of General Thoracic Surgery, Juntendo University School of Medicine, Tokyo, Japan

**Keywords:** lung cancer, sublobar resection, randomized trial, JCOG

## Abstract

Lobectomy and lymph node dissection have long been the standard surgical procedures for clinical stage IA lung cancer, based on a randomized trial published in 1995. That randomized trial showed better overall survival (OS) and recurrence-free survival (RFS) with lobectomy than with limited resection (segmentectomy or wide wedge resection) for clinical T1N0 non-small cell lung cancer. In addition, a three-fold higher rate of locoregional recurrence after limited resection was observed. However, recent advances in diagnostic imaging, such as thin-section computed tomography (TSCT), have improved the accuracy of clinical staging and the assessment of ground-glass opacity (GGO) in lung cancer, which is now recognized as being associated with less invasive pathology and a favorable prognosis. Subsequently, to evaluate the efficacy of sublobar resection for early-stage lung cancer, three major JCOG trials (JCOG0802/WJOG4607L, JCOG0804/WJOG4507L, and JCOG1211) were conducted. These studies stratified patients according to the consolidation-to-tumor ratio (CTR) on preoperative TSCT findings. These studies have recently disclosed their results, and they support the efficacy of sublobar resection, although several questions remain in daily clinical practice. In this perspective article, we summarize the current status and optimal surgical strategy for early-stage lung cancer based on these randomized controlled trials and discuss future perspectives, including the potential expansion of segmentectomy to larger or node-positive lung tumors.

## Introduction

**Figure 1:**
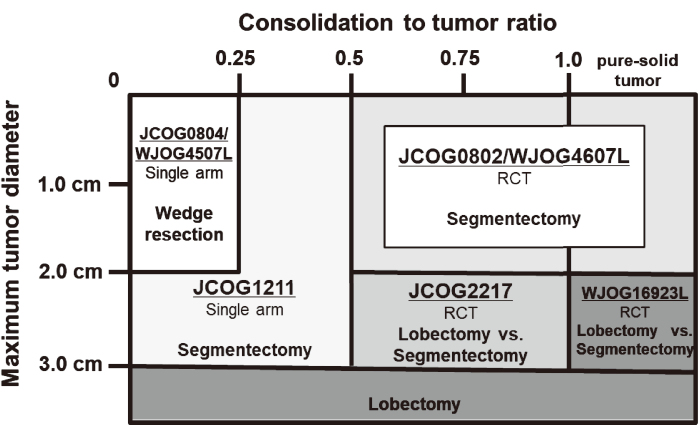
Surgical strategy map in the JOCG Lung Cancer Surgical Study Group

Since ‘radical lobectomy' was reported by Cahan in 1960^1)^, the standard surgical care for lung cancer has been lobectomy, which involves excising units of the lobe with their specific regional hilar and mediastinal lymphatics. However, pulmonary function-preserving limited resection for lung cancer has gradually became more prevalent in the late 20th century. In 1995, Ginsberg et al.^2)^ conducted a randomized controlled trial in which limited resection (segmentectomy and wide-wedge resection) and lobectomy for stage I lung cancer were compared. The results indicated that limited resection should not be applied to healthy patients with clinical stage IA lung cancer because it increased death rates, while local relapse rates were three-times higher^2)^. However, due to the advancement of thin-section computed tomography (TSCT) scans, the detection of small-sized and early-stage lung cancers has improved^3)^. Furthermore, the presence of ground-glass opacity (GGO) in lung nodules, as recognized on TSCT scan, has also been correlated with less-invasive pathological findings and favorable prognoses^4-9)^. Subsequently, the Lung Cancer Surgical Study Group of the Japan Clinical Oncology Group (JCOG) conducted a cohort study of early peripheral lung cancer. They investigated the validity of the TSCT criteria to diagnose non-invasive lung adenocarcinoma for the preoperative identification of pathological non-invasive cancer (JCOG0201)^5)^. Following this observational study, three important clinical trials were initiated by the JCOG (JCOG0802/WJOG4607L^10)^, JCOG0804/WJOG4507L^11)^, and JCOG1211^12)^) to evaluate the efficacy of sublobar resection for early-stage lung cancer based on tumor malignancy (Figure 1). The primary result of these studies have already been published and have greatly influenced daily practice. In the meantime, based on the results of clinical trials conducted by JCOG Lung Cancer Surgical Study Group, new randomized trials are ongoing to evaluate whether it is possible to apply segmentectomy as an expanded indication for larger tumors greater than 2 cm in size^13)^. In the future, these trials will clarify the significance of sublobar resection and uncover new insights to improve patient survival in lung cancer surgery.

In this perspective article, we report the current status and optimal surgical strategy for early-stage lung cancer based on the results of randomized controlled trials by JCOG. Furthermore, as a future perspective, we discuss the possibility of expanding the indications for segmentectomy.

### Indication of sublobar resection for GGO-dominant early-stage lung cancer

**Figure 2:**
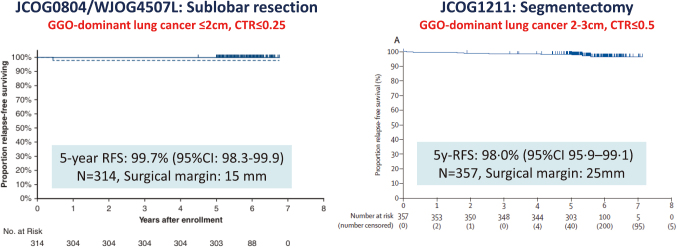
The primary results of JCOG0804/WJOG4507L and JCOG1211 trials

Following the result of JCOG0201^5)^, we started a trial of sublobar resection for peripheral early-stage lung cancer diagnosed by TSCT findings in 2008 (JCOG0804/WJOG4507L). This trial evaluated the efficacy of wide wedge resection for peripheral lung adenocarcinoma radiologically diagnosed as non-invasive, defined as a consolidation-to-tumor ratio (CTR) ≤ 0.25^11)^. This target enrollment of 333 patients was completed in 2011. The primary endpoint, 5-year relapse-free survival (RFS), was assessed in 314 patients who underwent sublobar resection. Operative modes were 258 wide wedge resections and 56 segmentectomies. The median pathological surgical margin was 15 mm (range, 0-55). The 5- year RFS was 99.7% (90% confidence interval (CI), 98.3-99.9), which met the primary end point, without any local relapse (Figure 2). Grade 3 or higher postoperative complications, based on the Common Terminology Criteria for Adverse Events (CTCAE) v3.0, were observed in 17 patients (5.4%); no grade 4 or 5 events occurred. Furthermore, the 10-year RFS for the 314 patients who underwent sublobar resection was 98.6% (95% CI: 96.2-99.5), though one local recurrence was observed at the resection margin. This trial concluded that peripheral GGO-dominant lung cancer can be cured by sublobar resection, with wedge resection considered the first choice.

Furthermore, based on several retrospective studies and the excellent long-term outcomes of patients with tumors ≤ 3 cm and CTR ≤ 0.5 in the JCOG0201 supplemental analysis^14)^, JCOG1211 was designed to assess the efficacy and technical feasibility of segmentectomy in clinical T1abN0 GGO- predominant non-small cell lung cancer (NSCLC) (CTR ≤ 0.5) with tumor diameters up to 3 cm^12)^. The primary endpoint was the 5-year RFS. Segmentectomy with hilar, interlobar, and intrapulmonary lymph node dissection was performed, and resection margins exceeding the maximum tumor diameter or at least 2 cm were mandatory. A total of 396 patients were registered, of whom 357 underwent segmentectomy. At a median follow-up of 5.4 years, the 5-year RFS was 98.0% (95% CI: 95.9-99.1) (Figure 2). The median pathological surgical margin was 25 mm (range, 17-32). Local relapse occurred only in one patient (0.3%; 95% CI: 0.0-1.4) among the 396 enrolled. Grade 3 or 4 early postoperative complications occurred in seven patients (2%), but no grade 5 treatment-related deaths occurred. Based on these results, segmentectomy should be considered part of the standard treatment for patients with predominantly GGO-type NSCLC ≤ 3 cm in diameter, including those with GGO components exceeding 2 cm. Together, these two trials demonstrate that GGO-dominant lung cancer is an optimal candidate for sublobar resection and that the optimal choice between wedge resection and segmentectomy should be determined by achievable surgical margin.

### Indication for segmentectomy in radiologically invasive lung cancer

**Figure 3:**
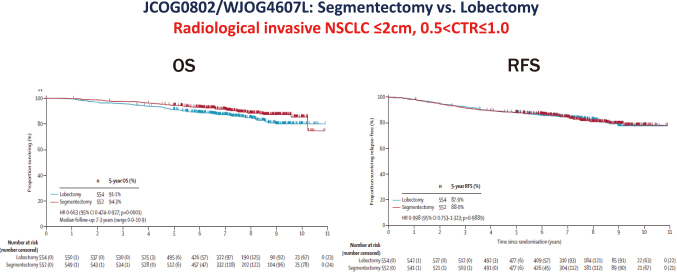
The primary results of JCOG0802/WJOG4607L and JCOG1211 trial

To evaluate the efficacy of segmentectomy or sublobar resection for peripherally located, small-sized NSCLC with a radiologically invasive appearance, two randomized phase III trials were conducted: one in Japan (JCOG0802/WJOG4607L)^10)^ and one in North America (CALGB140503)^15)^. In the JCOG and WJOG joint trial, the 5-year overall survival (OS) was 94.3% (95% CI: 92.1−96.0) after segmentectomy and 91.1% (95% CI: 88.4−93.2) after lobectomy. Both superiority and non-inferiority in OS were confirmed using a stratified Cox regression model (HR: 0.663; 95% CI: 0.474-0.927; one-sided p < 0·0001 for non-inferiority; p = 0·0082 for superiority) (Figure 3). At the 1-year follow-up, the difference in the reduction of median forced expiratory volume in 1 second between the groups was 3.5% (p < 0·0001), which did not reach the predefined threshold of 10% for clinical significance. The 5-year RFS was 88.0% (95% CI: 85.0−90.4) after segmentectomy and 87.9% (95% CI: 84.8−90.3) after lobectomy (HR, 0.998; 95% CI: 0.753−1.323; p = 0.9889) (Figure 3). Local relapse occurred in 10.5% of patients after segmentectomy and in 5.4% after lobectomy (p = 0.0018). Among patients who died, 52 of 83 (63%) after lobectomy and 27 of 58 (47%) after segmentectomy died from other diseases. No 30-day or 90-day mortality was observed. Furthermore, a phase III trial conducted in the USA demonstrated that sublobar resection was not inferior to lobectomy with respect to disease-free survival for peripheral stage IA NSCLC. Taken together, JCOG0802/WJOG4607L and CALGB140503 provide strong evidence for segmentectomy in clinical stage IA, peripherally located, small-sized NSCLC.

### Prevention of the locoregional recurrence for the optimal indication of segmentectomy

In JCOG0802/WJOG4607L, the survival benefit of segmentectomy is thought to be related to the preservation of postoperative pulmonary function, which may reduce deaths from other diseases or secondary cancers. Understanding the reason behind this survival advantage is therefore clinically important. One important reason is the higher rate of death from other diseases after lobectomy compared with segmentectomy. Furthermore, patients in the segmentectomy group were more frequently able to receive treatment for recurrent or second lung cancers, including a higher rate of repeat surgical resection. This observation implies that segmentectomy preserves physiological reserves, allowing for more intensive treatment of future life-threatening diseases, which improves long-term OS compared with lobectomy.

**Figure 4:**
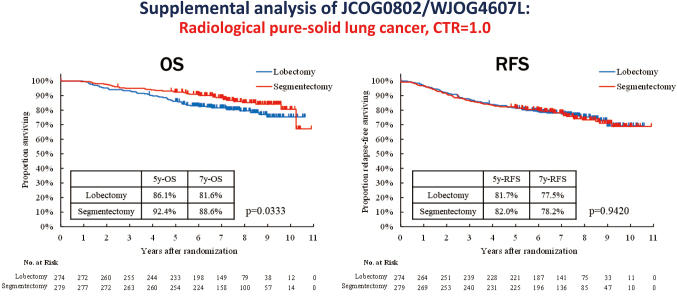
The results of JCOG0802/WJOG4607L supplemental analysis focusing on the radiological pure-solid NSCLC

Although segmentectomy demonstrated superior OS, the incidence of local relapse was higher after segmentectomy than that after lobectomy. In daily practice, radiologically determined pure-solid NSCLCs are known to exhibit more malignant behavior and have a worse prognosis than part-solid NSCLCs with a GGO component. In JCOG0802/WJOG4607L, however, the 5-year OS was significantly higher after segmentectomy than lobectomy in patients with radiologically pure-solid NSCLC (86.1% [95% CI: 81.4-89.7] vs. 92.4% [95% CI: 88.6-95.0]; HR: 0.64, 95% CI: 0.41-0.97; log-rank test p = 0.033), whereas the 5-year RFS was similar between the groups (81.7% [95% CI: 76.5-85.8] vs. 82.0% [95% CI: 76.9-86.0]; HR 1·01 [95% CI: 0.72-1.42]; p = 0.94)^10)^ (Figure 4). Since the exact mechanisms underlying this OS advantage remain unclear, a post-hoc supplemental analysis of JCO0802/WJOG4607L was performed to compare survival, cause of death, and recurrence patterns in patients with clinical stage IA, peripherally located, radiologically pure-solid NSCLC^16)^. The results indicated that death from other diseases was twice as common after lobectomy as that after segmentectomy (12.0% vs. 5.7%), whereas locoregional recurrence was twice as common after segmentectomy (16.1% vs. 7.7%, p = 0.0021). In particular, lymph node recurrence was three-fold higher after segmentectomy, and cut-end recurrence occurred exclusively in the segmentectomy group. In an additional supplemental analysis of 529 segmentectomy cases, multivariate analysis revealed two independent predictors of locoregional recurrence: a surgical margin smaller than the tumor size (p = 0.0063) and a radiological pure-solid tumor (p = 0.0008)^17)^.

**Figure 5:**
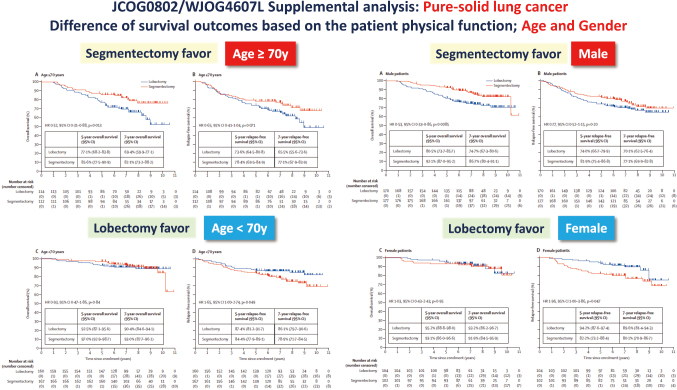
The subgroup analysis of JCOG0802/WJOG4607L supplemental analysis based on the patient age or gender among the patients with radiological pure-solid NSCLC

Importantly, survival outcomes of segmentectomy also differed greatly according to patient physiological function, including age and sex, in this post-hoc analysis. In subgroup analyses, better 5-year OS after segmentectomy than lobectomy was observed in the patients aged 70 years or older (77.1% [95% CI: 68.2-83.8] vs. 85.6% [95% CI: 77.5-90.9]; p = 0.013) and in male patients (80.5% [95% CI: 73.7-85.7] vs. 92.1% [95% CI: 87.0-95.2]; p = 0·0085). In contrast, better 5-year RFS after lobectomy was observed in patients younger than 70 years (87.4% [95% CI: 812-91.7] vs. 84.4% [95% CI: 77.9-89.1]; p = 0.049) and in female patients (94.2% [95% CI; 87.6-97.4] . 82.2% [95% CI: 73.2-88.4]; p = 0.047) (Figure 5). These results highlight the need for further research to refine the clinical indications for segmentectomy in radiologically pure-solid NSCLC and to determine the optimal balance between reduced invasiveness and effective cancer control by definitive resection^16)^.

### Future perspectives on sublobar resection as a treatment for larger tumors (> 2-3 cm) and node- positive lung cancer

**Figure 6:**
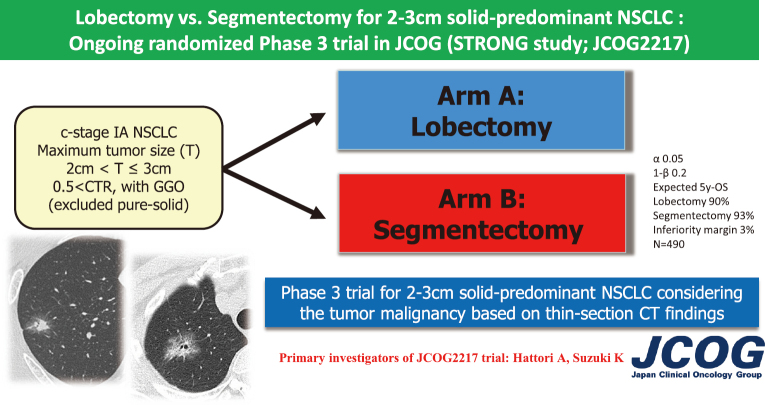
The schema of new randomized controlled trial in JCOG Lung Cancer Surgical Study Group (JCOG2217)

For T1cN0 NSCLC, lobectomy is the standard surgical mode. However, comparing segmentectomy with lobectomy in predominantly solid NSCLC (CTR > 0.5) with a tumor diameter of > 2 cm is necessary for expanding the indications for segmentectomy in the future. There are indications that predominantly solid tumors possess stronger malignancy characteristics. In particular, pure-solid tumors (CTR = 1.0 without any GGO) on TSCT are reported to have a worse prognosis than part-solid tumors (tumors with GGO)^18-29)^. A supplemental analysis of JCOG0201 showed that pure-solid tumors had a higher frequency of pathological nodal involvement and lower 5-year OS than the part-solid tumors, with this trend becoming more pronounced as clinical stage progresses (5-year overall survival in the GGO group: T1b 93.7% and T1c 91.7%; while in the solid group: T1b 85.9% and T1c 73.7%)^30)^. When pure-solid tumors were included, segmentectomy was inferior to lobectomy for the OS in lung cancers with > 2-cm and ≤ 3-cm tumor diameter according to several large-scale database and retrospective institutional studies in both the USA and Japan^31-34)^. In contrast, a retrospective study reported that when pure-solid tumors on TSCT were excluded, segmentectomy showed similar prognosis compared to lobectomy even in predominantly solid NSCLC with tumor diameter > 2 cm and ≤ 3 cm (5-year OS: 95.5% vs. 90.2%, p = 0.697)^35)^. Based on these results, we expected that segmentectomy would provide good prognosis for patients with predominantly solid NSCLC with GGO on TSCT and tumors > 2 cm and ≤ 3 cm in diameter. Consequently, we excluded pure-solid tumors from this trial because separately analyzing the treatment development for pure-solid tumors of > 2 cm would be more beneficial due to their high-grade malignancy. To date, in the JCOG Lung Cancer Surgical Study Group, a new randomized phase 3 trial is ongoing to confirm the efficacy of segmentectomy for predominantly solid lung cancer showing GGO in JCOG (JCOG2217; STRONG trial)^13)^ as the next clinical trial following JCOG0802/WJOG4607L (Figure 6).

Furthermore, another challenging discussion is arising in the general thoracic surgical community to consider the role of segmentectomy for node-positive lung cancer. In the JCOG0802/WJOG4607L supplemental analysis, the 5 year-OS of segmentectomy for NSCLC with occult nodal metastasis was superior to that of lobectomy in patients with node-positive lung cancer among a limited number of cases (n = 65, 5 year-OS; 94.4% vs. 78.7%, HR: 0.448, 5%CI: 0.130-1.544, p = 0.1919). Retrospectively, several nationwide or institutional studies have been conducted to evaluate the efficacy of segmentectomy for resectable NSCLC with unsuspected nodal disease. In the National Cancer Database study in the USA, segmentectomy showed equivalent 5 year- OS compared with lobectomy for both the N1 and N2 groups, and a multivariable model revealed that the use of adjuvant chemotherapy significantly improves survival in patients with lymph node metastasis (N1/N2) independent of the type of anatomic lung resection^36)^. Several other studies have demonstrated similar surgical outcomes for this population, suggesting segmentectomy had a favorable role and that complete lobectomy is unnecessary for occult node positive cases^37-41)^. Clearly, conversion to lobectomy is the mainstay for node- positive lung cancer when considering definitive locoregional management. However, segmentectomy may potentially improve survival even for this population. Notably, the JCOG0802/WJOG trial indicated a potential benefit of segmentectomy due to the effects of extensive treatment for subsequent life-threatening diseases including lung cancer recurrence or secondary disease^10)^. Further discussion is warranted to discuss the expanding indication of segmentectomy in the future.

## Conclusions

In this perspective article, we presented the latest clinical evidence and the operative strategies for small-sized lung cancers. In early-stage lung cancer, the presence of a GGO strongly indicates oncological aggressiveness and prognosis. According to these trials, GGO-dominant early-stage lung cancers may be considered optimal candidates for sublobar resection. For radiological invasive NSCLC, segmentectomy is a new standard option for surgical care, while further research is necessary to confirm the clinically relevant indications of segmentectomy for pure-solid NSCLC. To confirm the efficacy of segmentectomy for larger tumors, a new randomized phase 3 trial is ongoing for patients with predominantly solid lung tumors with GGO that is > 2 cm in size. In the future, we hope to expand the indication of segmentectomy effectively considering the surgical balance between low invasiveness and locoregional cancer control.

## Author contributions

AH conceptualized the manuscript, performed data curation, formal analysis, investigation, and writing of the original draft. KS, KT, TM, MF contributed to conceptualization of the manuscript and supervised, reviewed, and edited the original draft.

## Conflicts of interest statement

The authors declare that there are no conflicts of interest.
